# MyD88 Is a Critical Regulator of Hematopoietic Cell-Mediated Neuroprotection Seen after Stroke

**DOI:** 10.1371/journal.pone.0057948

**Published:** 2013-03-04

**Authors:** Catherine E. Downes, Connie H. Y. Wong, Katya J. Henley, Pedro L. Guio-Aguilar, Moses Zhang, Robert Ates, Ashley Mansell, Benjamin T. Kile, Peter J. Crack

**Affiliations:** 1 Department of Pharmacology, University of Melbourne, Parkville, Victoria, Australia; 2 The Walter and Eliza Hall Institute of Medical Research, Parkville, Victoria, Australia; 3 Department of Medical Biology, University of Melbourne, Parkville, Victoria, Australia; 4 Monash Institute of Medical Research, Monash University, Clayton, Victoria, Australia; University of Queensland, Australia

## Abstract

Neuroinflammation is critical in the neural cell death seen in stroke. It has been shown that CNS and peripheral responses drive this neuroinflammatory response in the brain. The Toll-like receptors (TLRs) are important regulators of inflammation in response to both exogenous and endogenous stressors. Taking advantage of a downstream adapter molecule that controls the majority of TLR signalling, this study investigated the role of the TLR adaptor protein myeloid differentiation factor 88 (*MyD88*) in the control of CNS and peripheral inflammation. Reversible middle-cerebral artery occlusion was used as the model of stroke *in vivo; in vitro* primary cultured neurons and glia were subject to four hours of oxygen and glucose deprivation (OGD). Both *in vitro* and *in vivo Myd88^−/−^* animals or cells were compared with wild type (WT). We found that after stroke *Myd88^−/−^* animals have a larger infarct volume compared to WT animals. Interestingly, *in vitro* there was no difference between the survival of *Myd88^−/−^* and WT cells following OGD, suggesting that peripheral responses were influencing stroke outcome. We therefore generated bone marrow chimeras and found that *Myd88^−/−^* animals have a smaller stroke infarct than their radiation naive counterparts if their hematopoietic cells are WT. Furthermore, WT animals have a larger stroke than their radiation naive counterparts if the hematopoietic cells are *Myd88^−/−^*. We have demonstrated that *MyD88*-dependent signalling in the hematopoietic cell lineage reduces infarct size following stroke and that infiltrating cells to the site of neuroinflammation are neuroprotective following stroke.

## Introduction

Myeloid differentiation factor-88 (MyD88) acts as an adaptor protein in the signalling of the Toll-Like receptors (TLRs) and the interleukin-1 (IL-1) receptor [1], [2]. These receptors detect pathogen associated molecular patterns (PAMPs), highly conserved regions among various pathogens. As well as PAMPs we now know that TLRs are capable of detecting endogenous damage associated molecular patterns (DAMPs). This study investigated whether MyD88-dependent signalling contributes to the damage associated with stroke.

Stroke induced hypoxia and ischemia causes cells directly affected to suffer energy failure and die. Upon doing so they release many intracellular components, several of which are TLR ligands, including heat shock proteins (HSPs), hyluronic acid, DNA complexes and heparin sulphate [3], [4]. The binding of TLRs leads to the activation of kinases, and subsequent activation of transcription factors including AP1 and NFκB [5]. These transcription factors then go on to cause the release of pro- and anti-inflammatory cytokines including, IL-1, IL-10, IL-8, IL-12 and chemokines including chemokine (C-C motif) ligand 2 (CCL-2) [6], [7], [8]. The inflammatory environment created due to TLR activation is proposed to be both beneficial and detrimental, in the case of stroke this is particularly true as dead and dying cells need to be removed and it is difficult for the resident phagocytotic cells, microglia, to manage excessive inflammation. Therefore understanding the contribution of a crucial signalling pathway critical to the pathogenesis of sterile cerebral inflammation is important.

It has recently been shown that TLR2 contributes to the inflammation following cerebral ischemia [9], but did not alter the attraction of granulocytes to the infarct area. Attraction of invading cells is an important component of stroke pathophysiology as they can both propagate and control inflammation in the brain [10], [11]. The release of IL-10 from T-regulatory cells has been shown to control the inflammatory response following stroke and can lead to a smaller infarct size [12]. TLRs are highly expressed on cells of a granulocytic origin and there is early evidence for TLR involvement in the recruitment of hematopoietic cells following CNS injury [13], [14]. The response to stroke is a complex integration of both the CNS and invading cells from the periphery. This study employed *MyD88* ^−/−^ animals and bone marrow chimeras to elucidate the role of MyD88-dependent signalling in both components of this integrated response.

## Methods

### Animals

All animal experiments complied with the regulatory standards of, and were approved by, the Walter and Eliza Hall Institute Animal Ethics Committee and the University of Melbourne, Medicine Dentistry and Health Sciences Animal Ethics Committee (Ethics #0911133). MyD88 −/− mice were on a C57Bl6 background, male and backcrossed to 15 generations and were generated by and kindly sourced from Professor Akira [6]. All surgery was performed under isoflurane anesthesia, and all efforts were made to minimize suffering.

### Primary glial culture

Primary glial cultures were isolated from 1 day old pups as described previously [15]. Briefly, cortices were isolated and meningies removed the clean cortices were digested in 0.02% v/v trypsin and mechanically dissociated until a single cell suspension was achieved. Cells were initially plated at a density of 1 brain in 10 ml of DMEM with 10% v/v FBS and 100 U/ml penicillin G, and 100 μg/ml streptomycin sulfate, media was changed every three days, after 7 days in culture cells were split with trypsin and thereafter when 85% confluence was reached. Glial cultures were used between 14 and 28 days *in vitro*.

### Primary neuron culture

Primary neurons were isolated as described previously [16]. Briefly, cortices of E14 pups were isolated and meningies removed, the clean cortices were digested in typsin A single cell suspension was ensured with mechanical disruption, cells were plated at a density of 6.0×10^5^ cells cm^2^ in neurobasal media containing 2% v/v B-27 supplement, 0.25% v/v 200 mM l-Glutamine, 0.1% v/v gentamicin and 10% v/v fetal bovine serum (FBS). After 3 hours all media was changed to FBS free media and thereafter every 3 days a half media changed was conducted. All experiments took place after 10–12 days in culture and neuronal purity was deemed to be at least 90% with NeuN (Neuronal Nuclei).

### Oxygen glucose deprivation

Both neurons and glia were plated on NUNC tissue culture plates and received a complete change of media to glucose free media (Gibco) at half the normal volume and were placed in the oxygen and glucose deprivation (OGD) chamber de-gassed for 5 minutes with 100% N_2_ and subsequently placed in a normal CO_2_ incubator for 4 hours. At the end of OGD glucose containing media (Gibco) was added to the cells to make up the normal volume. Control cultures received the same media changes with media containing glucose.

### MTT assay

24 hours after the completion of OGD 3-(4,5-dimethylthiazol-2-yl)-2,5-diphenyl-tetrazolium-bromide (MTT, Sigma) was added at 10% v/v to the culture media. This was incubated at 37°C for 45 minutes. Subsequently all media was removed, 200 μl of dimethyl sulfoxide (DMSO) was added and read for absorbance at 595 nm.

### Chimera development

Endogenous haematopoiesis was ablated in adult C57BL/6 CD45.1 or *Myd88^−/−^* mice by irradiation (11 Gy in 2 equal doses 2–3 hours apart). The heads of the mice were shielded. These recipient animals were then injected intravenously with 1×10^6^ unfractionated bone marrow cells from un-manipulated C57BL/6 CD45.1 or *Myd88^−/−^* mice. 8 weeks post-transplantation, chimerism was determined by flow cytometric analysis of peripheral blood leukocyte in conjunction with CD45-specific monoclonal antibodies. Only mice exhibiting donor engraftment of greater than 80% were used in this study.

### MCAO

Middle cerebral artery occlusion (MCAO) surgery was conducted as described previously [17] and modified [18]. All MCAO experiments and analysis were carried out in a randomised operator blinded fashion. Briefly, mice were anesthetised with 5% isoflurane in O_2_ and throughout surgery maintained on 2% isoflurane. The external carotid artery was isolated and whilst the common carotid artery was temporally clamped a silicone filament of 0.21±0.02 mm (Doccol) diameter was inserted via the external carotid to occlude the middle cerebral artery for a period of one hour. Occlusion was monitored by laser doppler flowmetry and greater than 75% occlusion was deemed sufficient, which all mice achieved. To end occlusion the mice were re-anesthetized and the filament was withdrawn. Throughout and following surgery mice were maintained at 37°C. There was no surgical mortality WT mice and 7% in *MyD88−/−* mice. At 24 hours of reperfusion mice were killed and brains were immediately removed and sliced in a mouse brain matrix to 500 µ m thickness. These were placed in a 2% 2,3,5-Triphenyltetrazolium chloride (TTC) in PBS solution at 35°C for 15 minutes. Photomicrographs were captured using a Zeiss Axioskop microscope and infarct area was determined using the Image J software (v1.47; NIH). Tissue swelling in the injured side was accounted for by dividing the infarct area from each section by the ratio of the areas of the injured relative to injured side. The Cavalieri formula was used to calculate total lesion volume [Volume  =  ΣA x t x ISF] where A = sum of the corrected infarct areas; t = section thickness (500 µm) and ISF = inverse of the sampling fraction.

### Evaluation of neurological deficits

Neurological deficits of the mice that had undergone stroke surgery were measured on a scale of 1–4 [17], 24 h after surgery. The following grading system was used: 1, normal spontaneous movements; 2, animal unable to extend fore paw; 3, animal circling toward left; and 4, animal crouched and unresponsive to noxious stimuli.

### Immunofluorescence and Infiltrate detection analysis

At 24 hours of reperfusion animals were anesthetised as above and tissue was fixed with a transcardial perfusion with 4% Para-formaldehyde in PBS. Brains were the cut into 10 μm sections and mounted on superfrost plus glass microscope slides. For immunofluorescence coronal sections were blocked in CAS-block (Invitrogen) for 45 min at room temperature and then exposed to primary antibody overnight at 4C. The primary antibody targeting NeuN (Millipore) was used at 1∶1000 and Mac-1 [19] was used at 1∶10 in a 1% w/v solution of BSA in PBS. Sections were then washed with TBS-t for 3×15 min and incubated with secondary antibody for 45 min at room temperature. Secondary antibodies were used at a dilution of 1∶1000in a 1% w/v solution of BSA in PBS (Alexa-fluor 488 and 595 anti rat and mouse, Invitrogen). Following secondary antibody incubation slides were was in TBS-T for 3×15 min and cover slips mounted using a mounting media containing DAPI (Vectorshield-Vector) and images recorded on a Leica DMI 6000B fitted with wide field fluorescence. Leukocytes were detected with a naphthalene granulocyte assay (Sigma) as per manufacturers instructions. An optical fractionator stereological design [20] was employed to make unbiased estimates of Mac-1 positive cells in the infarcted cortex of WT, MyD88−/− and chimeric mice using Stereo Investigator software version 7. Cells that were positive to Mac-1 were counted on approximately 10 sections separated by 100 µm from each animal using a ×20 objective and a 200 µm ×150 µm frame area.

### Statistical analysis

All statistics were completed on Prism software, for multiple comparisons the means of the experimental groups were compared using the nonparametric Kruskal-Wallis test with a post-hoc Dunn’s multiple comparison test. The nonparametric Mann-Whitney test was used for comparison between two groups. P<0.05 was considered statically significant all data is displayed as the mean ± standard error of the mean (SEM).

## Results

### MyD88 signalling affects infarct size following MCAO

**Figure 1:**
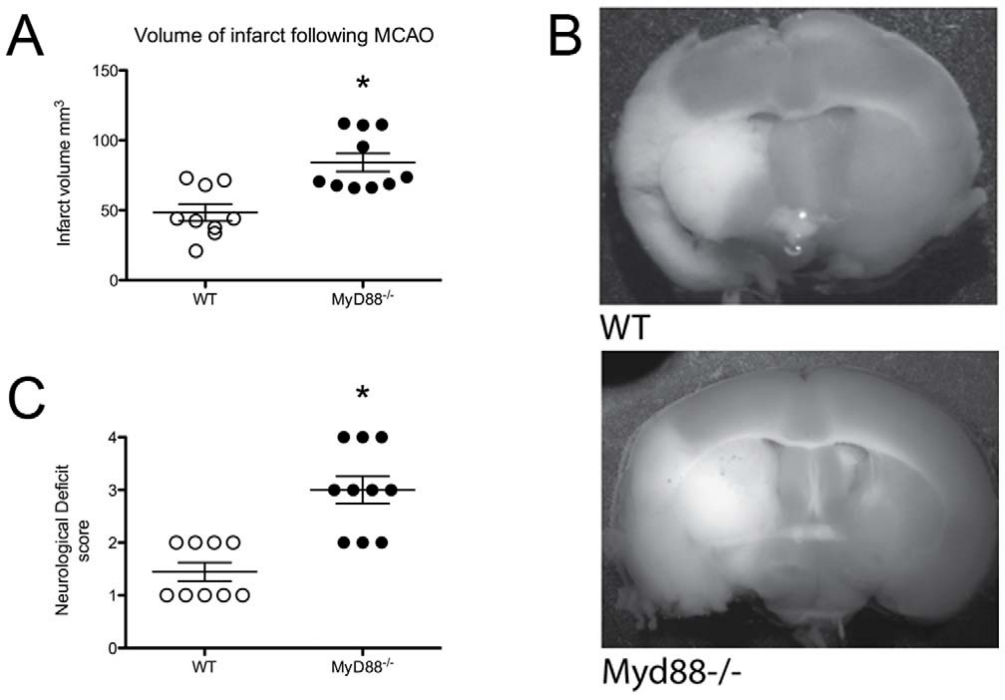
MyD88 influences infarct after stroke. A. *Myd88^−/−^* mice have a significantly larger volume of infarct than their WT counterparts (n = 9–10, * Significantly different from WT mice, *P*<0.05, error bars indicate ±SEM). B. Representative TTC staining demonstrates that the infarct in both the WT and *Myd88^−/−^* animals covers both the cortex and striatal regions of the brain. C. Neurological deficit wt (n = 9) and *Myd88^−/−^* (n = 10) mice. Scattergram shows the difference between the two groups. Animals were scored after 24 h of ischemia/reperfusion using the following criteria: 1, normal spontaneous movement; 2, unable to extend forepaw; 3, animal circling towards left; and 4, animal unresponsive to noxious stimuli.

The contribution of MyD88-dependent signalling to the inflammatory progression of infarct following stroke was assessed using the MCAO model of stroke. Following 2 hours of MCAO and 24 hours of reperfusion the *Myd88* ^−/−^ mice suffered a significantly larger infarct volume, WT 48.38±6.10 mm^3^ Vs. *Myd88* ^−/−^ 84.23±6.50 mm^3^ P<0.05, P = 0.0076 (Figure 1A), despite a similar drop in blood flow to the area of infarct during occlusion (Figure S1A). The infarct in both of the animals was spread across both the cortex and the striatum (Figure 1B). The measurement of body weight, mean arterial blood pressure, blood pH, PCO_2_ and O_2_ saturation were found to be not significantly different between genotypes and consistent with previously published data [18], [21] (supplementary data, table S1).

### MyD88 signalling does not affect neuronal survival

**Figure 2:**
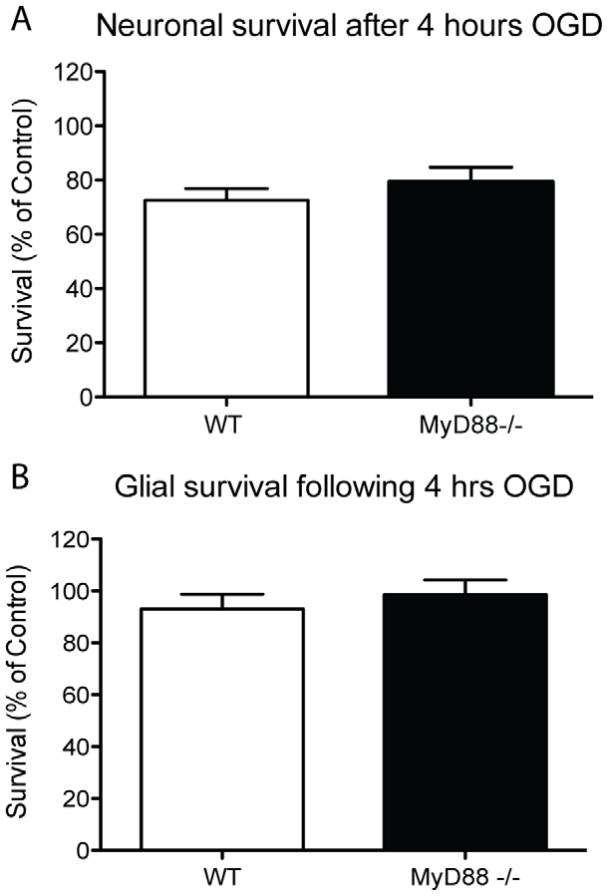
MyD88 does not influence the outcome in primary cultured neurons and glia after OGD. A & B. following 4 hours of OGD and 24 hours of reperfusion, there was no difference in the percentage of survival of WT and *Myd88^−/−^* primary neurons or glia as measured by MTT assay (n = 13).

To determine if the larger infarct size in *Myd88* ^−/−^ mice after stroke is due to the intrinsic response of the neurons and glia both WT and *Myd88* ^−/−^ primary cultured neurons and glia were subjected to 4 hours of oxygen and glucose deprivation (OGD). After 24 hours of reperfusion the survival of both cell types was assessed by MTT assay. The survival of neurons following 4 hours of OGD and 24 hours of reperfusion was the same regardless of the absence or presence of MyD88; this was also true for the WT and *Myd88* ^−/−^ glial cultures (Figure 2 A & B).

### MyD88 is required for cellular infiltration following MCAO

**Figure 3:**
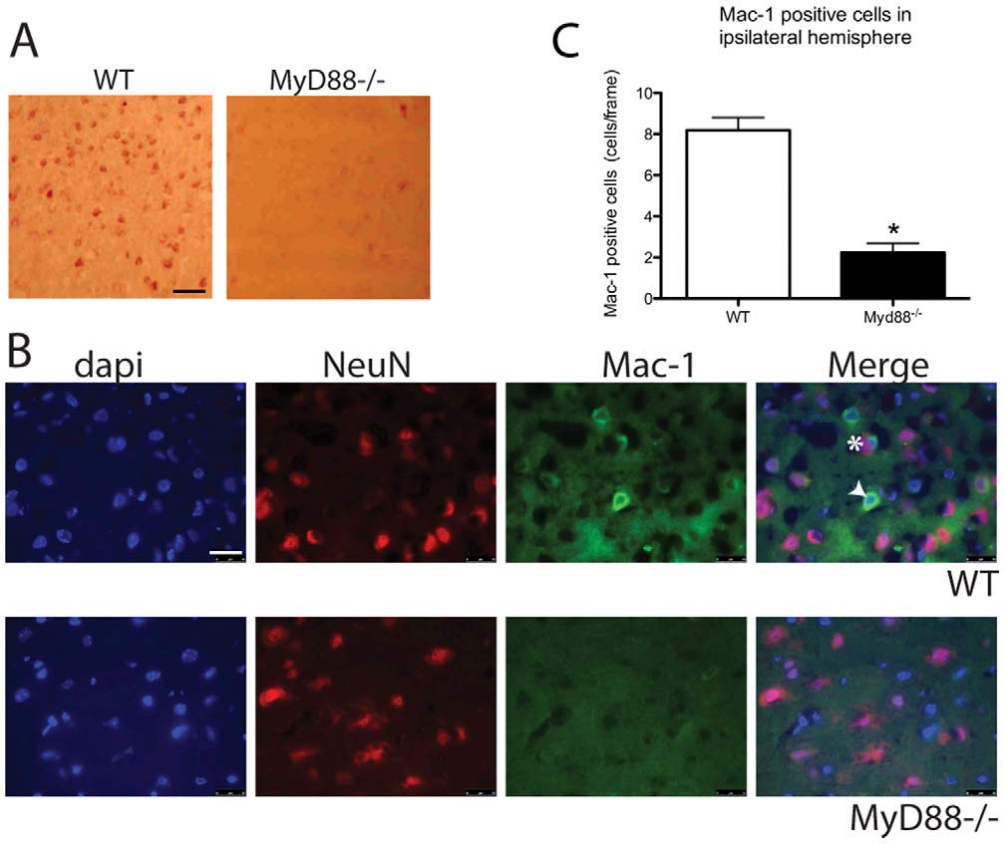
MyD88 is involved in cellular recruitment after stroke. A. The WT ipsilateral hemispheres display more cells positive for leukocyte esterases than the *Myd88^−/−^*, scale bar  = 50 µm . B. Mac-1 fluorescence revealed that cells positive for Mac-1 alone (arrowhead) and those positive for both NeuN and Mac-1 (hash) were identifiable in the WT ipsilateral hemisphere but not the *Myd88^−/−^ ,* scale bar  = 25 µm. C. Quantification of Mac-1 positive cells revealed significantly more Mac-1 positive cells in the WT compared to the *Myd88^−/−^* ipsilateral hemisphere (n = 5, * Significantly different from WT mice, *P*<0.05, error bars indicate ±SEM).).

To investigate the role of MyD88 in the attraction of haematopoietic cells to the injury site, WT and *Myd88^−/−^* brain sections were stained with a granulocyte specific esterase assay. WT mice brain sections demonstrate more leukocyte positive esterase cells than *Myd88* ^−/−^ animals (Figure 3A). Sections were further stained for Mac-1, as Mac-1 is present on activated microglia, macrophages, and neutrophils, its detection within injured tissue is likely to be indicative of an inflammatory response. Mac-1 was detected alone and co-localised with the neuronal nuclear marker NeuN in the WT mice (Figure 3B), primary antibody alone and secondary antibody showed no staining (data not shown). The few cells that were Mac-1 positive in the *Myd88* ^−/−^ mice were negative for NeuN. Both mice showed no Mac-1 positive cells on the contralteral side of the brain (data not shown). The number of Mac-1 positive cells per frame in the WT ipsilateral hemisphere was significantly higher than the number in the *Myd88* ^−/−^ brains (Figure 3C, WT 8.35±0.72 cells/frame Vs. *Myd88* ^−/−^ 2.42 0.52 cells/frame *P*<0.05). The WT showed more Mac-1 positive cells than the *Myd88* ^−/−^, and in the WT brains the Mac-1 co-localised with the neuronal marker NeuN. This is indicative of infiltrating phagocytic cells targeting neurons.

### MyD88 signalling in hematopoietic cells affects infarct size

**Figure 4:**
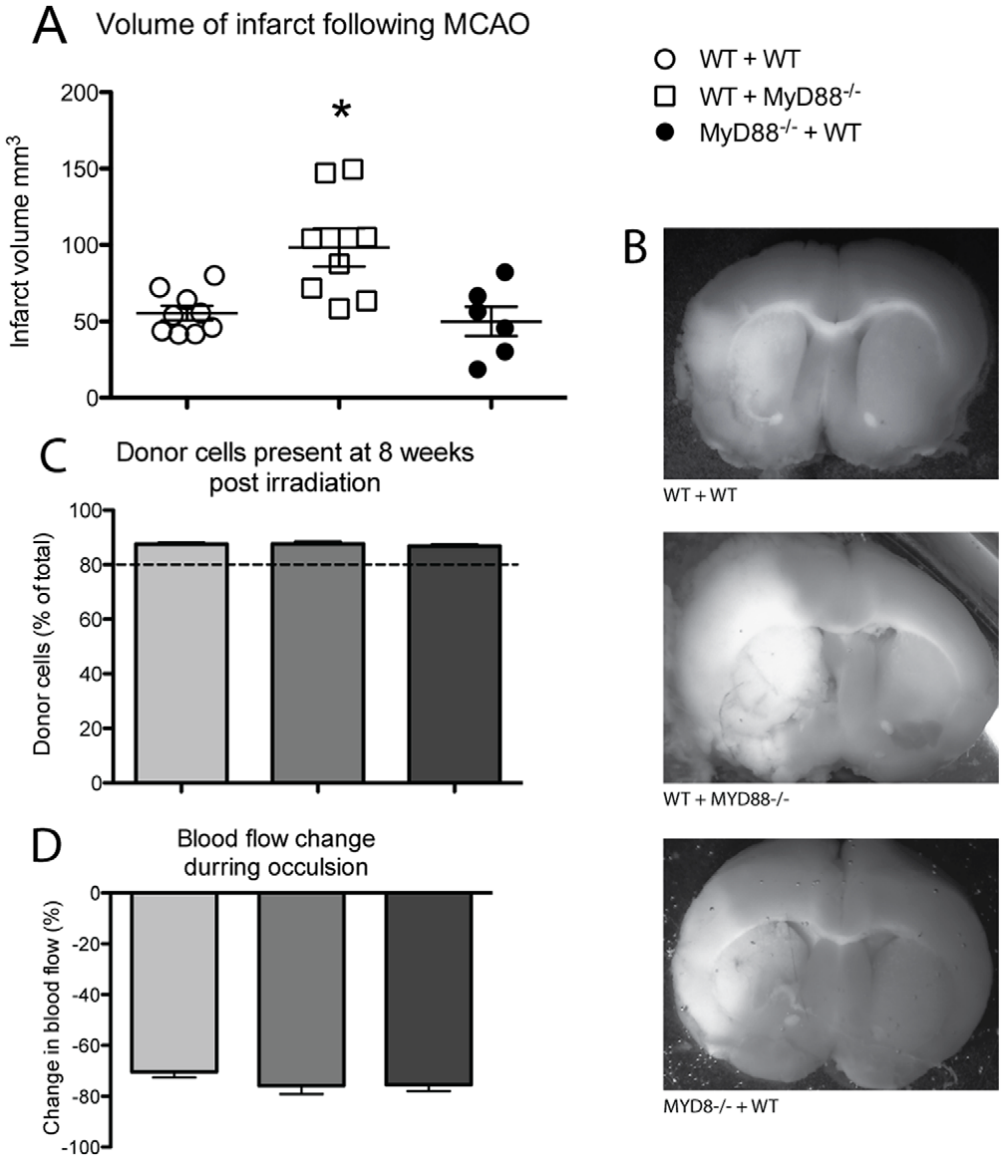
MyD88 influences the response of hematopoietic cells after stroke. A. Following 1 hour of MCAO and 24 hours of reperfusion the infarct area in all chimera groups covers both the cortical and striatal regions. B. When the infarct volume is calculated from the TTC stained tissue the WT + *Myd88^−/−^* groups shows a larger infarct volume than the WT + WT groups, whilst there is no difference between the *MyD88^−/−^* + WT and the WT + WT infarct volume (n = 9). All chimera mice had above 80% donor cells present (C, n = 32–45) and there was no difference in percentage decrease in blood flow between the groups (D, n = 21–34 * Significantly different from WT + WT mice, *P*<0.05 error bars indicate ±SEM).

The results shown in figure 2 were suggestive that the detrimental response to the stroke in the *Myd88^−/−^* was not due to intrinsic defects to the brain cells. This suggested that peripheral cells, namely hematopeotic cells were contributing to the outcome. To address this issue we generated chimera mice, deficient in Myd88 except cells of hematopoietic origin (*Myd88* ^−/−^ + WT), only in cells of hematopoietic origin (WT + *Myd88* ^−/−)^ or neither (WT + WT). All mice used in this study exhibited at least 80% donor engraftment at the time of the MCAO surgery, which was performed 8 weeks post-transplantation (Figure 4C). There was no significant difference in infarct volume between the WT radiation naive and WT mice that were reconstituted with WT haematopoietic cells (Figure. S1B). All of the chimera groups experienced a similar level of blood flow decrease at the time of occlusion, indicating a similar level of hypoxia and decrease in perfusion in all animals (Figure 4D). TTC staining demonstrated that infarcts in all groups were spread across both the cortex and striatum (Figure 4A). The WT + *Myd88* ^−/−^ mice had a significantly larger infarct volume than the WT + WT mice. The role of *Myd88* in the hematopoietic cells was further highlighted by the similar infarct volume between the WT + WT mice and the *Myd88* ^−/−^ + WT cells (Fig. 4A and B).

### MyD88 signalling in the CNS contributes to haematopoietic cell infiltration

**Figure 5:**
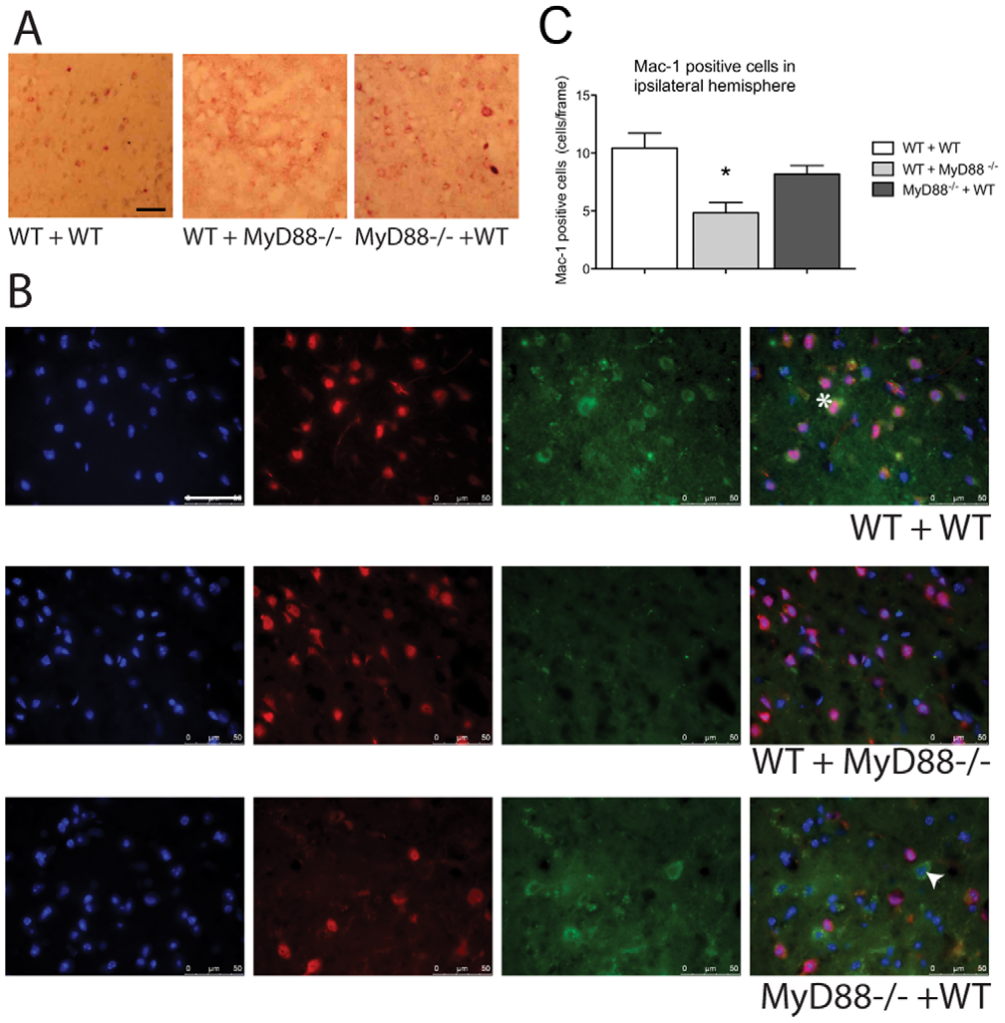
MyD88 mediates hematopoietic cell influence in the outcome after stroke. The WT + WT and *Myd88^−/−^* + WT ipsilateral hemispheres display more cells positive for leukocyte esterases, images representative of n = 3. B. The WT + WT chimera mice show co-expression of NeuN and Mac-1 following 24 hours of reperfusion in the ipsilateral side of the brain highlighted by the arrow. There is no expression of Mac-1 detectable in the WT + *Myd88^−/−^* chimeras. The *Myd88^−/−^* + WT chimeras show Mac-1 expression in the brain, however co-localisation with NeuN is minimal an example of a Mac-1 positive NeuN negative cell is highlighted with a hash, images representative of n = 3. C. The quantification of Mac-1 positive cells per frame revealed that there was significantly less Mac-1 cells in the WT + *Myd88^−/−^* ipsilateral hemispheres when compared to the WT + WT animals, scale bar  = 50 µm (n = 5, * Significantly different from WT + WT mice, *P*<0.05 error bars indicate ±SEM).

In the chimera studies the WT + WT and the *Myd88* ^−/−^ + WT display more cells positive for leukocyte esterase’s (Figure 5A). Mac-1 positive cells were identified in all chimera ipsilateral hemispheres, however there was significantly fewer Mac-1 positive cells in the WT + *Myd88* ^−/−^ compared to the WT + WT mice (WT + WT 9.93±1.42 cells/frame Vs. WT + *Myd88* ^−/−^ 4.8±1.31 cells/frame, *P*<0.05). There was no significant difference in the numbers of cells present between the WT + WT and the *Myd88* ^−/−^ + WT mice. However, the WT + WT Mac-1 positive cells were often co-localised with NeuN positive cells, whilst in the *Myd88* ^−/−^ this was rarely the case (Figure 5B). The WT + *Myd88* ^−/−^ mice show very few Mac-1 positive cells, whilst the *Myd88* ^−/−^ + WT sections demonstrate Mac-1 positive cells that are rarely also NeuN positive. This data indicates that Myd88-dependent signalling is required in the CNS to attract infiltrating cells to the injury site.

## Discussion

As an adaptor protein for primarily pro-inflammatory pathways we hypothesized that Myd88-dependent signalling would contribute to brain damage following stroke, and that Myd88^−/−^ mice would exhibit a smaller infarct following MCAO. In direct contrast, our study demonstrates that Myd88-dependent signalling in hematopoietic cells has a protective effect in stroke.

The major finding from this study is that hematopoietic cells exhibit a neuroprotective function after stroke that is mediated by MyD88. Other studies have demonstrated that invading cell numbers are protective [22], [23], [24] following stroke. These studies showed a decrease rather than a complete ablation of cellular infiltration. It is likely that infiltrating cells can have both beneficial and detrimental effects, and their relative number may contribute to this [25]. Interestingly, it has been reported that the removal of CCL-2 caused a decrease in the numbers of invading cells seen after stroke, which was mirrored by a corresponding increase in the activation of microglia, highlighting some form of coordinated response between endogenous and exogenous cell types in the brain [26]. One particular invading cell type that has been shown to be neuro-protective following stroke is the regulatory T cell (T-reg). The release of IL-10 from T-regs has been shown to decrease inflammation and a subsequent decrease in infarct volume after stroke [27]. Reducing the infiltration of all cells in to the infarct area will also decrease the numbers of T-regs, and may over the longterm prolong inflammation. Modulating gross numbers of cells attracted to the infarct area may be less important than influencing the types of hematopoietic cells and at what time of reperfusion they arrive.

Our findings concerning infarct size differ from those of Yang *et* *al.*, who found no difference in infarct size, oedema volume or neurological score between *Myd88* ^−/−^ and WT mice following MCAO [28]. However, in Yang’s study the MCA was occluded for 6 hours, an unusually long occlusion for the intraluminal suture/filament method. This extended period reportedly caused the infarct to cover more than 55% of the prosencephalon, a very large infarct size. It is likely that in both WT and *Myd88* ^-/-^ mice the infarct had covered the entire ipsilteral hemisphere and therefore the damage was so severe that no difference could be detected. The model used in the current study utilized a 1-hour period of MCA occlusion. This is sufficient to produce a moderate infarct, with significant regions of undamaged and at risk tissue present in the ipsilateral cortex. We believe this approach allows the detection of more subtle changes in infarct size. Our data contrasts with that of Famakin *et* *al.*, who found no difference in infarct size between *Myd88* ^−/−^ and WT mice in a permanently occlusive model [29]. This is perhaps unsurprising given that in the permanent occlusion model used by Famakin *et* *al.*, there was no tissue reperfusion. Given that our results indicate a protective role for hematopoietic cells that requires Myd88 and is supported by our finding that WT mice with MyD88^−/−^ haematopoietic cells suffered a larger infarct size than WT mice with WT haematopoietic cells, regardless of the genotype of the brain. This finding implicates the cells that invade from the periphery in the development of the infarct size following stroke, and shows that MyD88-dependent signalling in the invading cells in particular can control in the size of the infarct. Therefore in the model used by Famakin et al, a model that minimises invading cells access to the brain by excluding reperfusion, one would be expected to find little difference between the WT and MyD88^−/−^ infarct volume.

Using a similar model of stroke others have demonstrated a primarily deleterious role for TLR signalling in stroke [9], [30], [31]. IL-1 signalling has also been shown through the use of release inhibition and receptor blockade to be similarly deleterious [32]. An explanation for the apparently disparate results between select TLR knockouts and the results reported here maybe found in the complexities of TLR and IL-1 signalling. TLR signalling is often described as either MyD88-dependent or MyD88 independent, a shift in the balance by removing MyD88, as opposed to removing or inhibiting just one TLR would affect all TLRs dependent on MyD88. In the inflammatory environment following cerebral infarction ligands for multiple TLRs are present. MyD88 dependent signalling following LPS stimulation has been shown to activate mitogen activated kinase phosphotases (MAPKP). These phosphotases are important negative regulators of many inflammatory pathways [33]. Therefore the absence of MyD88 dependent signalling may lead to less negative regulation of signal transduction pathways, and MyD88-independent pathways may lead to the activation of pro-inflammatory mediators unimpeded.

The role of both TLR and IL-1 signalling following stroke is complex as the response to CNS inflammation and not only involves resident cells but those from the periphery. These numerous cell types, belonging to disparate systems have variable expression of the receptors involved in Myd88-dependent signalling, adding to the complexity of the response. By isolating the expression of Myd88 to either the CNS or the hematopoietic cells this study has made it possible to understand the contributions of MyD88-dependent signalling pathways within a two separate systems. The activation and release of cytokines, attraction and infiltration of hematopoietic cells to the CNS and the subsequent infarct volume are all influenced by activation of MyD88-dependent signalling. This study has demonstrated a role for MyD88-dpendent signalling in the response to MCAO further work to understand the location and particular pathways involved will significantly extend the field of neuro-immuno-biology.

## Supporting Information

Figure S1 **A. Lack of MyD88 causes no alteration in blood flow during MCAO. B.** Generation of bone marrow chimeras has no effect on MCAO outcome.
(TIF)

Table S1 (DOCX)
