# Supplementary figures and images for: MyD88 Is a Critical Regulator of Hematopoietic Cell-Mediated Neuroprotection Seen after Stroke
Source: PLoS One. 2013 Mar 4;8(3):e57948. doi: 10.1371/journal.pone.0057948 (PMC3587572; doi:10.1371/journal.pone.0057948)

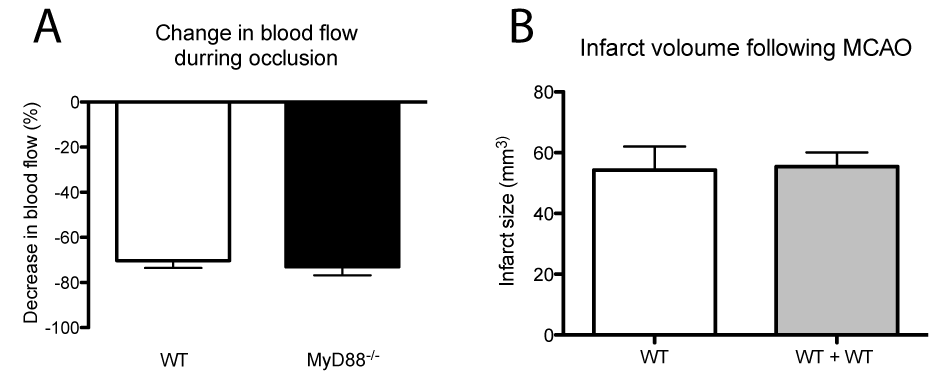

Supplement: Figure S1 — A. Lack of MyD88 causes no alteration in blood flow during MCAO. B. Generation of bone marrow chimeras has no effect on MCAO outcome. (TIF) [file pone.0057948.s001.tif]
