# Supplementary material for: MyD88 Is a Critical Regulator of Hematopoietic Cell-Mediated Neuroprotection Seen after Stroke
Source: PLoS One. 2013 Mar 4;8(3):e57948. doi: 10.1371/journal.pone.0057948 (PMC3587572; doi:10.1371/journal.pone.0057948)
Supplement: Table S1 — (DOCX) [file pone.0057948.s002.docx]

Supplemental table 1.

Arterial blood gas analysis

| Genotype | pH | pO_2_ | pCO_2_ |
| --- | --- | --- | --- |
| Wild type | 7.31 ± 0.05 | 105 ± 13.2 | 46.1 ± 4.7 |
| MyD88 -/- | 7.36 ± 0.07 | 102 ± 8.6 | 48.3 ± 3.8 |

Each value represents mean ± SEM (n=3).
